# Recent uptake of intermittent preventive treatment during pregnancy with sulfadoxine–pyrimethamine is associated with increased prevalence of *Pfdhfr* mutations in Bobo-Dioulasso, Burkina Faso

**DOI:** 10.1186/s12936-017-1695-1

**Published:** 2017-01-23

**Authors:** Mamoudou Cisse, Gordon A. Awandare, Alamissa Soulama, Halidou Tinto, Marie-Pierre Hayette, Robert T. Guiguemdé

**Affiliations:** 1 Laboratory of Parasitology and Entomology, Centre MURAZ, 01 BP 390, Bobo-Dioulasso, Burkina Faso; 2 West African Centre for Cell Biology of Infectious Pathogens, College of Basic and Applied Sciences, University of Ghana, Accra, Ghana; 3 Department of Clinical Research, Centre MURAZ, Bobo-Dioulasso, Burkina Faso; 4 Laboratory of Clinical Microbiology, University Hospital of Liège, Liège, Belgium

**Keywords:** Malaria, Pregnancy, Sulfadoxine–pyrimethamine, Drug resistance, Burkina Faso

## Abstract

**Background:**

The impact of sulfadoxine–pyrimethamine (SP) used as intermittent preventive treatment during pregnancy (IPTp-SP) on mutant parasite selection has been poorly documented in Burkina Faso. This study sought first to explore the relationship between IPTp-SP and the presence of mutant parasites. Second, to assess the relationship between the mutant parasites and adverse pregnancy outcomes.

**Methods:**

From September to December 2010, dried blood spots (DBS) were collected during antenatal care visits and at delivery from 109 pregnant women with microscopically confirmed *falciparum* malaria infection. DBS were analysed by PCR–restriction fragment length polymorphism (PCR–RFLP) for the polymorphisms at codons 51, 59, 108, and 164 of the *Pfdhfr* gene and codons 437 and 540 in the *Pfdhps* gene.

**Results:**

Both the *Pfdhfr* and *Pfdhps* genes were successfully genotyped in 92.7% (101/109) of the samples. The prevalence of *Pfdhfr* mutations N51I, C59R and S108N was 71.3, 42.6 and 64.4%, respectively. Overall, 80.2% (81/101) of samples carried the *Pfdhps* A437G mutation. None of the samples had the *Pfdhfr* I164L and the *Pfdhps* K540E mutations. The prevalence of the triple mutation N51I + C59R + S108N was 25.7% (26/101). The use of IPTp-SP was associated with a threefold increased odds of *Pfdhfr* C59R mutation [crude OR 3.29; 95% CI (1.44–7.50)]. Pregnant women with *recent* uptake of IPTp-SP were at higher odds of both the *Pfdhfr* C59R mutation [adjusted OR 4.26; 95% CI (1.64–11.07)] and the *Pfdhfr* intermediate-to-high resistance, i.e., ≥ 2 *Pfdhfr* mutations [adjusted OR 3.45; 95% CI (1.18–10.07)]. There was no statistically significant association between the presence of the *Pfdhfr* intermediate-to-high resistance and parasite densities or both maternal haemoglobin level and anaemia.

**Conclusion:**

The data indicate that despite the possibility that IPTp-SP contributes to the selection of resistant parasites, it did not potentiate pregnancy-associated malaria morbidity, suggesting the continuation of SP use as IPTp in Burkina Faso.

**Electronic supplementary material:**

The online version of this article (doi:10.1186/s12936-017-1695-1) contains supplementary material, which is available to authorized users.

## Background

Malaria during pregnancy persists as a major public health challenge with adverse consequences for mother and developing fetus [1]. The World Health Organization (WHO) has recommended since 2004 relevant strategies, such as the administration of intermittent preventive treatment with sulfadoxine–pyrimethamine during pregnancy (IPTp-SP), the use of insecticide-treated nets and the effective management of clinical cases to reduce the burden of malaria and improve pregnancy outcomes [2]. Current policy dictates that SP should be provided to mothers at each scheduled focused antenatal care (ANC) visit in the second and third trimesters [3]. Although IPTp-SP has shown considerable benefits to mother and fetus [4], the emergence of *Plasmodium falciparum* resistance to SP can jeopardize this strategy [5].

SP resistance is linked to point mutations in the parasite genome, specifically the *P. falciparum* dihydrofolate reductase (*Pfdhfr*) and dihydropteroate synthetase (*Pfdhps*) genes [6, 7]. Mutations in *Pfdhfr* confer resistance to pyrimethamine while mutations in *Pfdhps* confer resistance to sulfadoxine and other sulfa drugs [8]. The more the mutations, the stronger the resistance. The *Pfdhfr* triple mutant (N51I + C59R + S108N) and *Pfdhps* double mutant (A437G + K540E) have been strongly associated with potential resistance in sub-Saharan Africa [9–12].

The impact of IPTp-SP on selection for drug-resistant parasites in the field is not well understood due to reported contradictory results. Indeed, although IPTp-SP has been associated with the selection of mutations [10, 13–15], it has been shown that self-reported use of IPTp-SP did not increase the prevalence of resistant alleles [5, 16]. Moreover, conflicting results about the impact of mutant parasites on pregnancy outcomes such as parasite densities, maternal haemoglobin level, and anaemia have been reported [14, 15].

In Burkina Faso, IPTp-SP was adopted in 2005 and resistance to SP in pregnant women has been only reported in rural areas [17, 18], and none of those studies had assessed the impact of SP on mutant parasites selection. Nevertheless, Coulibaly et al. [17] have shown that SP remained highly effective in Ziniaré, with a PCR corrected failure rate of only 1.3% at 42 days, and a PCR uncorrected failure rate of 6.5%. This study sought first, to explore the relationship between IPTp-SP and the presence of mutant parasites. Second, to assess the relationship between the mutant parasites and adverse pregnancy outcomes, including parasite densities, maternal haemoglobin level, and anaemia in Bobo-Dioulasso, the second largest city of the country.

## Methods

### Study area, subjects and sample collection

This study used archived samples collected from two malaria-in-pregnancy studies conducted in Bobo-Dioulasso from September to December 2010. The study protocol was approved by the National Ethics Committee for Health Research of Burkina Faso. Written informed consent was obtained from all study participants.

Details for these two cross-sectional studies including samples collection have been described elsewhere [19, 20]. Briefly, during these studies, all pregnant women presenting for routine ANC or delivery were consecutively recruited at the primary health facilities of Kua and Lafiabougou (both located in the peri-urban area of Bobo-Dioulasso). Sociodemographic data and information about the current and previous pregnancies were documented. Iron and folate supplementation and SP prophylaxis were given free of charge to all women attending ANC at both health facilities. The uptake of the drugs was recorded in their antenatal care files, and information on the use SP prophylaxis was obtained from the pregnant women through questionnaire and from these files.

**Fig. 1:**
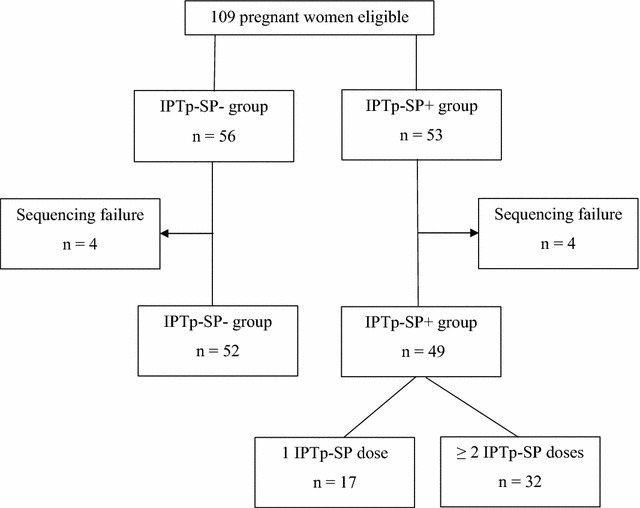
Flow chart of participants through the study

Based on these records, the women were classified as IPTp-SP+ if they took at least a first course of IPTp-SP and had correctly followed prophylaxis (Fig. 1), or IPTp-SP− group (control) if they had not received IPTp-SP since the beginning of their pregnancy. A finger-prick blood sample was collected from each participant for preparation of blood smears and blood spots on filter paper (Whatman grade 3). Maternal haemoglobin (Hb) concentration was measured using a haemoglobinometer (HemoCue AB, Angelhom, Sweden).

### Laboratory procedures

Molecular genotyping was performed only on samples from women with a positive blood slide. All molecular tests were performed at the West African Centre for Cell Biology of Infectious Pathogens, Department of Biochemistry, Cell and Molecular Biology, University of Ghana, Accra.

*Plasmodium falciparum* DNA was extracted from dried blood spots using QIAamp DNA Mini Kit 50 (QIAgen, USA) according to the manufacturer’s recommendations. Eluted DNA was immediately used in amplification reactions or was stored at −20 °C until further processing.

SP resistance-mediating single nucleotide polymorphisms were analysed in both *Pfdhfr* and *Pfdhps* genes using polymerase chain reaction (PCR) followed by restriction enzyme digestion as previously described [21]. Polymorphisms investigated were as follows: N51I, C59R, S108N, and I164L for *Pfdhfr* gene and A437G and K540E for *Pfdhps* gene. Nested PCR products were resolved by 2.5% gel electrophoresis and results classified as wild type, pure mutant, and mixed infection (presence of both wild type and pure mutant in the same sample) on the basis of differential band sizes.

### Definitions and statistical analyses

Data were double entered in Excel 2013 and analyses performed using STATA 12 (Stata Corp, College Station, TX, USA).

Mixed infection was considered as mutant. Infections were defined as intermediate-to-high resistance of *Pfdhfr* (≥2 *Pfdhfr* mutations) if *Pfdhfr* double and *Pfdhfr* triple mutations were detected [13]. Pregnant women were classified into primigravida (first-time mothers) and multigravida (those with at least one previous pregnancy). Age was stratified as ≤20 and >20 years. *Recent* IPTp-SP uptake was defined as receipt of the last SP dose within 2.5 months of blood sample collection, and *early* IPTp-SP uptake if the interval between last SP dose and the blood sample collection was >2.5 months. Anaemia was defined as an Hb level lower than 11.0 g/dL [13]. Parasite density values were log-transformed for statistical analyses.

Proportions for categorical variables were compared using Pearson’s Chi Square test. Comparisons of means between groups were done by the Student’s t test, while medians were compared by using the Wilcoxon rank-sum test.

Logistic regression models were estimated to evaluate factors associated with the intermediate-to-high resistance of *Pfdhfr* and maternal anaemia. Linear regression models were used to assess predictors of log-transformed parasite densities and maternal Hb. Multivariable analyses were built using backward stepwise regression models, with an inclusion criterion of *P* value < 0.05 and exclusion criterion p value > 0.10. Statistical significance was set for a *P* value < 0.05.

## Results

### Characteristics of study participants

Maternal age, residence and education status were comparable between both IPTp-SP+ and IPTp-SP− groups. However, primigravida were more frequent in the IPTp-SP− group (*P* = 0.05) compared to the IPTp-SP+ group (Table 1). No statistically significant differences were found between the IPTp-SP groups in both maternal Hb levels and proportion with anaemia. However, the median parasite density in the IPTp-SP+ group (4080 parasites/μL) was higher than that in the IPTp-SP− group (2100 parasites/μL), although this difference was not statistically significant (*P* = 0.06).

**Table 1:** Baseline characteristics of the study participants

Characteristic	Total (N = 101)	IPTp-SP− (n = 52)	IPTp-SP+ (n = 49)	*P* value
Mean age (years, ±SD)	22.8 ± 5.0	22.1 ± 4.6	23.5 ± 5.3	0.16^a^
Age group				0.90^a^
≤20	45 (44.5)	23 (44.2)	22 (44.9)	
>20	56 (55.5)	29 (55.8)	27 (55.1)	
Education				0.53^a^
No formal schooling	67 (66.3)	33 (63.5)	34 (69.4)	
Formal schooling	34 (33.7)	19 (36.5)	15 (30.6)	
Gravidity				0.05^a^
Primigravida	75 (74.3)	43 (82.7)	32 (65.3)	
Multigravida	26 (25.7)	9 (17.3)	17 (34.7)	
Residence				0.10^a^
Lafiabougou	43 (42.6)	18 (41.9)	25 (58.1)	
Kua	58 (57.4)	34 (58.6)	24 (41.4)	
Median of parasite/μL (IQR)	2960 (5120)	2100 (4140)	4080 (11,080)	0.06^b^
Mean haemoglobin (g/dL), ±SD	10.3 ± 1.7	10.4 ± 1.7	10.1 ± 1.7	0.30^c^
Anaemia (% Hb < 11 g/dL)	64 (36.6)	31 (40.4)	33 (32.7)	0.40^a^

^a^Pearson Chi square Test

^b^ Wilcoxon rank-sum test

^c^ Student’s t test

### Relationship between IPTp-SP use and the prevalence of *Pfdhfr* and *Pfdhps* mutations

Overall 101 out of 109 parasite isolates (92.7%) were successfully genotyped for both *Pfdhfr* and *Pfdhps* genes (Fig. 1). The prevalence of the *Pfdhfr* N51I, C59R and S108N mutations was 71.3, 42.6 and 64.4%, respectively. No mutation in the *Pfdhfr* gene at codon 164 was detected in any of the parasite isolates. Pregnant women who took the IPTp-SP had lower odds of the *Pfdhfr* N51I mutation compared to those who did not use any IPTp-SP [crude OR 0.24; 95% CI (0.09–0.62)]. The IPTp-SP+ group had greater than threefold increased odds of *Pfdhfr* C59R mutation compared to the IPTp-SP− group [crude OR 3.29; 95% CI (1.44–7.50)] (Table 2). In multivariable analysis, pregnant women with *recent* uptake of IPTp-SP were at higher odds of carrying the *Pfdhfr* C59R mutation compared to the *early* IPTp-SP group [adjusted OR 4.26; 95% CI (1.64–11.07)]. An additional table shows this in more detail (see Additional file 1: Table S1). Considering the *Pfdhfr* gene alone, the double mutation (including N51I + C59R, N51I + S108N or C59R + S108N) was the most prevalent combination, observed in 40.6% (41/101) of the isolates. The triple mutation N51I + C59R + S108N was found in 25.7% (26/101) of the isolates. The triple mutation was more predominant in the IPTp-SP+ group (32.6%) than in the IPTp-SP− group (19.2%), however the difference was not statistically different (*P* = 0.1). With regard to *Pfdhps* gene 80.2% (81/101) of samples carried the A437G mutation. The proportions of the A437G mutation in both IPTp-SP groups were statistically similar (*P* = 0.7). No *Pfdhps* K540E mutation was observed in any of the isolates tested.

**Table 2:** Prevalence of *Pfdhfr* and *Pfdhps* mutations according to IPTp-SP groups

Mutation allele	Total (N = 101)	IPTp-SP− (n = 52)	IPTp-SP+ (n = 49)	Crude OR 95% CI	*P* value
*Pfdhfr* N51I					0.003
No	29 (28.7)	8 (15.4)	21 (42.9)	1	
Yes	72 (71.3)	44 (84.6)	28 (57.1)	0.24 (0.09–0.62)	
*Pfdhfr* C59R					
No	58 (57.4)	37 (71.2)	21 (42.9)	1	0.005
Yes	43 (42.6)	15 (28.9)	28 (57.1)	3.29 (1.44–7.50)	
*Pfdhfr* S108N					
No	36 (35.6)	22 (42.3)	14 (28.6)	1	0.1
Yes	65 (64.4)	30 (57.7)	35 (71.4)	1.83 (0.80–4.20)	
*Pfdhps* A437G					
No	20 (19.8)	11 (21.2)	9 (18.4)	1	0.7
Yes	81 (80.2)	41 (78.8)	40 (81.6)	1.19 (0.45–3.19)	
*Pfdhfr* double mutation					0.7
No	60 (59.4)	30 (57.7)	30 (61.2)	1	
Yes	41 (40.6)	22 (42.3)	19 (38.8)	0.86 (0.40–1.91)	
*Pfdhfr* triple mutation					0.1
No	75 (74.3)	42 (80.8)	33 (67.4)	1	
Yes	26 (25.7)	10 (19.2)	16 (32.6)	2.04 (0.82–5.07)	
≥2 *Pfdhfr* mutation					0.3
No	34 (33.7)	20 (38.5)	14 (28.6)	1	
Yes	67 (66.3)	32 (61.5)	35 (71.4)	1.56 (0.69–3.60)	

Overall, 66.3% (67/101) of the isolates carried two or more *Pfdhfr* mutations, classified as the *Pfdhfr* intermediate-to-high resistance. The prevalence of infections with at least 2 *Pfdhfr* mutations was more predominant in the IPTp-SP+ group (71.4%) than in the IPTp-SP− group (61.5%), however the difference was not statistically different (*P* = 0.3) (Table 2). Nevertheless, further analysis showed that the uptake of one dose of IPTp-SP was significantly associated with the prevalence of infections with at least 2 *Pfdhfr* mutations [adjusted OR 5.34; 95% CI (1.10–26.40)]. An additional table shows this in more detail (see Additional file 2: Table S2). In addition, in multivariable logistic regression, pregnant women with *recent* uptake of IPTp-SP were at higher odds of the *Pfdhfr* intermediate-to-high resistance compared to those who did not use any IPTp-SP [adjusted OR 3.45; 95% CI (1.18–10.07)] (Table 3). Early receipt of IPTp-SP had lower odds of the *Pfdhfr* intermediate-to-high resistance compared to those who did not use any IPTp-SP [adjusted OR 0.61; 95% CI (0.20–1.86)] although this association did not reach statistical significance.

**Table 3:** Risk factors associated with the *Pfdhfr* intermediate-to-high resistance

Variable	N	≥2 *Pfdhfr* mutations (%)	Adjusted OR (95% CI)^a^	*P* value
Time between last SP dose and the survey				
No IPTp-SP	52	32 (61.5)	1	
*Early* IPTp-SP	19	10 (52.6)	0.61 (0.20–1.86)	0.38
*Recent* IPTp-SP	30	25 (83.3)	3.45 (1.18–10.07)	0.023

^a^Multivariable analysis using logistic regression adjusted for residence, age, and gravidity

### Effect of the *Pfdhfr* intermediate-to-high resistance on maternal outcomes

There was no statistically significant association between the presence of the *Pfdhfr* intermediate-to-high resistance and parasite densities, maternal Hb level and prevalence of maternal anaemia (Tables 4, 5). However, significant predictors for high parasite densities included primigravidae and *early* IPTp-SP receipt (Table 4). Furthermore, living in the area of the primary health centre of Kua resulted in a significant increase of 0.90 g/dL in the maternal Hb level [adjusted regression coefficient = 0.90; 95% CI (0.24–1.55)] (Table 4) and a lower odds of anaemia [adjusted OR 0.35; 95% CI (0.14–0.84)] (Table 5).

**Table 4:** Predictors of parasite density and maternal haemoglobin level

Maternal outcome	Adjusted coefficient (95% CI)	*P* value
Log10 of parasite density (parasite/μl)		
Primigravida	0.35 (0.08 to 0.62)^a^	0.011
*Early* IPTp-SP	0.30 (−0.01 to 0.60)^a^	0.05
Haemoglobin level (g/dl)		
Living in Kua	0.90 (0.24 to 1.55)^b^	0.008

^a^Multivariable analysis using linear regression adjusted for residence, age, and *Pfdhfr* intermediate-to-high resistance

^b^ Multivariable analysis using linear regression adjusted for age, gravidity, education, use of IPTp-SP, parasite density, and *Pfdhfr* intermediate-to-high resistance

**Table 5:** Risk factors associated with maternal anaemia

Variable	N	Anaemia (%)	Adjusted OR (95% CI)^a^	P value
Residence				
Lafiabougou	43	33 (76.7)	1	
Kua	58	31 (53.5)	0.35 (0.14–0.84)	0.018

^a^Multivariable analysis using logistic regression adjusted for age, gravidity, education, use of IPTp-SP, parasite density, and *Pfdhfr* intermediate-to-high resistance

## Discussion

This study analysed samples collected 5 years after adoption of IPTp-SP in Burkina Faso. The prevalence of *Pfdhfr* triple mutation N51I + C59R + S108N in the present study (25.7%) was low compared to the rates of 44.9 and 50% reported in pregnant women at the same period in Ziniaré [17] and in the general population [22], respectively. However, Tahita et al. [18] reported a lower prevalence of 11.4% in pregnant women during the same period in Nanoro, a small town in Burkina Faso. Those studies had been carried out in rural settings where malaria transmission is high and seasonal, mainly occurring during the months of August–December [17, 18, 22]. In Burkina Faso, SP had been used as second-line treatment before 2005, but was increasingly used as a first-line drug after chloroquine was discontinued in 2005, when artemisinin combination therapy (ACT) was not yet readily available [23, 24]. Therefore, those different reports suggest either a variation in SP pressure according to study areas or differing access to ACT [18]. The prevalence of *Pfdhfr* triple mutation reported in this study was also lower than those previously reported during pregnancy in other African countries where the proportion of *Pfdhfr* triple mutation ranged from 36 to 75% [5, 8, 13, 14, 16, 25]. The *Pfdhps* A437G mutation is very common across Africa [16, 26] and its prevalence in this study (80.2%) was higher than the reported rates (34.2–75.3%) in other studies conducted during the same period in pregnant women from Burkina Faso [17, 18]. This difference could be attributed to a variation in the level of SP use in different parts of the country, and the effect of widespread use of an antibiotic drug, namely cotrimoxazole in the population [27]. Indeed, this mutation is involved in resistance to sulfadoxine in endemic areas and its selection by SP in pregnant women has been recently shown in Cameroon [26]. Both the *Pfdhfr* I164L and the *Pfdhps* K540E mutations were not detected in the present study, suggesting that these are still absent in Burkina Faso. This is consistent with previous reports in the country [17, 18, 22, 23, 28, 29] as well as in other settings in West Africa [8, 13, 14, 25]. However, a low proportion of *Pfdhps* K540E mutation (0.37%) has been recently reported in Mali [17].

In accordance with previous reports from Ghana [13] and Mali [30], this study showed that the *Pfdhfr* N51I mutation was not selected by the use of IPTp-SP. However, the use of IPTp-SP was associated with an increased prevalence of the *Pfdhfr* C59R mutation. Significantly, IPTp-SP was associated with an increased prevalence of both the *Pfdhfr* C59R mutation and *Pfdhfr* intermediate-to-high resistance when SP was expected to be present in peripheral blood, i.e., if the last IPTp-SP dose was taken within 2.5 months before the blood collection. Furthermore, among pregnant women who benefited from one dose of IPTp-SP, more than 70% of them had used IPTp-SP recently. This is a plausible explanation of the association between the uptake of one dose of IPTp-SP and the prevalence of infections with at least 2 *Pfdhfr* mutations. This is the first study directly implicating the *recent* use of IPTp-SP in the selection of drug resistant parasite mutants in pregnant women from Burkina Faso. The findings from this study are consistent with those from previous studies carried out in Mozambique [10] and in Tanzania [15], where it was shown that *recent* receipt of SP was associated with greater prevalence of resistant parasites. This suggests that these resistant parasites are no longer competitive during re-infection with other strains and that resistance decreases when active drug pressure decreases [10, 31]. Several observational studies in Africa had shown the association between the use of IPTp-SP and the increased prevalence of mutations [13, 14, 26, 32]. However, those studies did not use the timing between the last dose of IPTp-SP and the blood sample collection as a proxy to further assess this association.

Higher parasite densities were frequent in pregnant women with early receipt of IPTp-SP compared to those who did not use any IPTp-SP. After stratification by the presence of *Pfdhfr* intermediate-to-high resistance, this association was statistically significant in primigravida who harboured parasites with low *Pfdhfr* grade of resistance. This is not surprising because parasite densities decrease due to acquired immunity against malaria parasites when parity increases [13]. The use of IPTp-SP was not associated with higher parasite densities in Mozambique [10] and in Malawi [11], despite the possibility that IPTp-SP contributes to the selection of quintuple mutant-resistant parasites. Those findings contrast with a report from Tanzania where *recent* receipt of SP was associated with higher parasite densities due to the presence of *Pfdhps* mutation at codon 581 in that study [15].

This study showed that the *Pfdhfr* intermediate-to-high resistance was not associated with maternal anaemia and maternal Hb level. This is consistent with previous reports from Mozambique and Malawi [10, 11]. A plausible explanation is the lack of an effect of the mutations on parasite densities [10]. Nevertheless, living in the area of the primary health centre of Kua was a protecting factor against anaemia. Malarial anaemia is usually associated with more prolonged infections [33], therefore, it is likely that women living nearer to the health centre sought medical attention sooner and thus avoided the development of anaemia.

The limitations of this study include the small size of the studied population and the cross-sectional approach used. A longitudinal study would enable a better assessment of the impact of SP on the selection of mutant parasites by comparing the level of drug resistance before and after SP use.

## Conclusion

The present study provides an update on the prevalence of mutations conferring SP resistance 5 years after the implementation of the IPTp-SP policy in Burkina Faso. SP given for IPTp selects for both *Pfdhfr* C59R mutation and *Pfdhfr* intermediate-to-high mutant parasites in maternal peripheral blood when SP is still present in blood. However *Pfdhfr* intermediate-to-high resistance is not associated with pregnancy-associated malaria morbidity. Altogether, the findings from this study suggest that SP may still be efficacious when used as IPTp. Nevertheless, further studies are needed to evaluate the in vitro and in vivo efficacy of SP.

## Additional files

**Additional file 1: Table S1.** Risk factors associated with the *Pfdhfr* C59R mutation. The data provided showed the association between time between last SP dose and the survey and the prevalence of *Pfdhfr* C59R mutation using a logistic regression model adjusted for residence, age, and gravidity.

**Additional file 2: Table S2.** Association between the number of SP doses and the *Pfdhfr* intermediate-to-high resistance. The data provided showed the association between the number of SP doses and the prevalence of at least 2 *Pfdhfr* mutations using a logistic regression model adjusted for residence, age, and gravidity.

## Abbreviations

ACT: artemisinin combination therapy
ANC: antenatal clinic
CI: confident interval
DBS: dried blood spots
Hb: haemoglobin
IPTp-SP: intermittent preventive treatment with sulfadoxine–pyrimethamine
OR: odd ratio
*Pfdhfr*: *P. falciparum* dihydrofolate reductase
*Pfdhps*: *P. falciparum* dihydropteroate synthetase
RFLP: restriction fragment length polymorphism
WHO: World Health Organization
