# Supplementary material for: Recent uptake of intermittent preventive treatment during pregnancy with sulfadoxine–pyrimethamine is associated with increased prevalence of Pfdhfr mutations in Bobo-Dioulasso, Burkina Faso
Source: Malar J. 2017 Jan 23;16:38. doi: 10.1186/s12936-017-1695-1 (PMC5259838; doi:10.1186/s12936-017-1695-1)
Supplement: Supplementary file 1 — Additional file 1: Table S1. Risk factors associated with the Pfdhfr C59R mutation. The data provided showed the association between time between last SP dose and the survey and the prevalence of Pfdhfr C59R mutation using a logistic regression model adjusted for residence, age, and gravidity. [file 12936_2017_1695_MOESM1_ESM.docx]

**Table S1 Risk factors associated with the *Pfdhfr* C59R mutation**

| Variable | N | *Pfdhfr* C59R mutation (%) | Adjusted OR (95% CI)^h^ | *P*-value |
| --- | --- | --- | --- | --- |
| Time between last SP dose and the survey |  |  |  |  |
| No IPTp-SP | 52 | 15 (28.9) | 1 |  |
| *Early* IPTp-SP | 19 | 9 (47.4) | 2.22 (0.75-6.55) | 0.15 |
| *Recent* IPTp-SP | 30 | 19 (63.3) | 4.26 (1.64-11.07) | 0.003 |

^h^ Multivariable analysis using logistic regression adjusted for residence, age, and gravidity.
