# Supplementary material for: Recent uptake of intermittent preventive treatment during pregnancy with sulfadoxine–pyrimethamine is associated with increased prevalence of Pfdhfr mutations in Bobo-Dioulasso, Burkina Faso
Source: Malar J. 2017 Jan 23;16:38. doi: 10.1186/s12936-017-1695-1 (PMC5259838; doi:10.1186/s12936-017-1695-1)
Supplement: Supplementary file 2 — Additional file 2: Table S2. Association between the number of SP doses and the Pfdhfr intermediate-to-high resistance. The data provided showed the association between the number of SP doses and the prevalence of at least 2 Pfdhfr mutations using a logistic regression model adjusted for residence, age, and gravidity. [file 12936_2017_1695_MOESM2_ESM.docx]

**Table S2 Association between the number of SP doses and the *Pfdhfr* intermediate-to-high resistance**

| Variable | N | ≥2 *Pfdhfr* mutations (%) | Adjusted OR (95% CI)^i^ | *P*-value |
| --- | --- | --- | --- | --- |
| SP doses |  |  |  |  |
| 0 | 52 | 32 (61.5) | 1 |  |
| 1 | 17 | 15 (88.2) | 5.34 (1.10-26.40) | 0.04 |
| ≥2 | 32 | 20 (62.5) | 0.78 (0.29-2.10) | 0.61 |

^i^ Multivariable analysis using logistic regression adjusted for residence, age, and gravidity.
